# Parallel Microbial Ecology of *Pasteuria* and Nematode Species in Scottish Soils

**DOI:** 10.3389/fpls.2019.01763

**Published:** 2020-01-28

**Authors:** Jamie N. Orr, Roy Neilson, Thomas E. Freitag, David M. Roberts, Keith G. Davies, Vivian C. Blok, Peter J. A. Cock

**Affiliations:** ^1^Cell and Molecular Sciences, The James Hutton Institute, Dundee, United Kingdom; ^2^School of Life and Medical Sciences, University of Hertfordshire, Hatfield, United Kingdom; ^3^Ecological Sciences, The James Hutton Institute, Dundee, United Kingdom; ^4^Division of Biotechnology and Plant Health, Norwegian Institute of Bioeconomy Research, Ås, Norway

**Keywords:** metabarcoding, nematode, *Pasteuria*, biocontrol, ecology

## Abstract

*Pasteuria* spp. are endospore forming bacteria which act as natural antagonists to many of the most economically significant plant parasitic nematodes (PPNs). Highly species-specific nematode suppression may be observed in soils containing a sufficiently high density of *Pasteuria* spp. spores. This suppression is enacted by the bacteria *via* inhibition of root invasion and sterilization of the nematode host. Molecular methods for the detection of *Pasteuria* spp. from environmental DNA (eDNA) have been described; however, these methods are limited in both scale and in depth. We report the use of small subunit rRNA gene metabarcoding to profile *Pasteuria* spp. and nematode communities in parallel. We have investigated *Pasteuria* spp. population structure in Scottish soils using eDNA from two sources: soil extracted DNA from the second National Soil Inventory of Scotland (NSIS2); and nematode extracted DNA collected from farms in the East Scotland Farm Network (ESFN). We compared the *Pasteuria* spp. community culture to both nematode community structure and the physiochemical properties of soils. Our results indicate that *Pasteuria* spp. populations in Scottish soils are broadly dominated by two sequence variants. The first of these aligns with high identity to *Pasteuria hartismeri*, a species first described parasitizing *Meloidogyne ardenensis*, a nematode parasite of woody and perennial plants in northern Europe. The second aligns with a *Pasteuria*-like sequence which was first recovered from a farm near Edinburgh which was found to contain bacterial feeding nematodes and *Pratylenchus* spp. encumbered by *Pasteuria* spp. endospores. Further, soil carbon, moisture, bulk density, and pH showed a strong correlation with the *Pasteuria* spp. community composition. These results indicate that metabarcoding is appropriate for the sensitive, specific, and semi-quantitative profiling of *Pasteuria* species from eDNA.

## Introduction

Plant parasitic nematodes (PPNs) pose a major threat to global food security, with estimated combined crop losses due to PPNs equating to $80 billion USD each year (Nicol et al., 2011; Jones et al., 2013). However, broad spectrum chemical nematicides and soil fumigants which have been effective in PPN control are being steadily withdrawn from the market due to environmental concerns, such as ozone depletion by methyl bromide (Ristaino and Thomas, 1997), or their potential for negative effects on human health, such as the 1985 Aldicarb poisoning outbreak in the United States and Canada (Goldman et al., 1990). The withdrawal of these substances necessitates the development of sustainable alternatives to chemical PPN control. These alternatives may be: management practices, such as solarization (McGovern and Mcsorley, 1997; Freitas et al., 2000; Wang et al., 2006), or incorporation of organic amendments (Stirling et al., 2003; Jaffee, 2004; Walker, 2004; Bonanomi et al., 2007); breeding and growth of resistant cultivars, such as potato varieties which carry the *H1* resistance gene, preventing the development of *Globodera rostochiensis via* starvation of the infective juvenile stages within the root (Kort et al., 1977; Rice et al., 1985; Sobczak et al., 2005); and the incorporation or cultivation of PPN biocontrol agents (BCAs) in cropping soils, such as the nematophagous fungus *Pochonia chlamydosporia*, which parasitizes the eggs of some of the most impactful PPN species (Kerry et al., 1982; Yang et al., 2012; Manzanilla-López et al., 2013). Among BCAs specialist nematode parasites are the most effective as generalist nematode predators do not respond to large increases in PPN populations providing only a background level of biocontrol which may not be easily quantified (Stirling, 2014). Among the most specialized PPN BCAs are obligate hyperparasitic bacteria of the genus *Pasteuria*.

*Pasteuria* spp. are gram positive, endospore forming Firmicutes which suppress PPNs *via* two mechanisms. First, *Pasteuria* spp. endospores attach to the surface of the nematode hindering directional movement and, by extension, root access (Davies et al., 1991; Vagelas et al., 2012). Second, upon penetration of the nematode cuticle and colonization of the pseudocoelom, *Pasteuria* spp. are able to alter embryogenesis, sterilizing the host (Davies et al., 2011). *Pasteuria* spp. may be highly fastidious parasites, exhibiting host specificity which can be species or population specific (Davies et al., 2001; Davies et al., 2008; Duneau et al., 2011; Mohan et al., 2012). Cross generic attachment profiles have been described in *Pasteuria* spp. which are capable of attachment to both the pigeon pea cyst nematode (*Heterodera cajani*) and a potato cyst nematode (*G. pallida*) (Mohan et al., 2012), however these are both members of the Heteroderinae with similar life cycles. Specificity of *Pasteuria* spp. presents an advantage over broad spectrum chemical control and less targeted management practices such as soil solarization which may remove ecosystem services that are mediated by beneficial organisms, including BCAs (Wang et al., 2006). However, this host specificity also presents a challenge to the use of *Pasteuria* spp. as inundative or inoculative BCAs, as the interaction of a strain with a native PPN population cannot be easily predicted without prior testing. Inoculative and conservation biocontrol using *Pasteuria* spp. is hindered by a limited understanding of the impacts of soil properties and management practices on *Pasteuria* spp. populations.

oil characteristics, such as clay and organic matter content have been noted as a driver of *Pasteuria* biology (Dabiré and Mateille, 2004; Dabiré et al., 2007). Spores are non-motile and so require a degree of porosity in the soil in order to disperse and to come into contact with the nematode cuticle, allowing attachment and infection (Dabiré and Mateille, 2004). *Pasteuria* spp. endospores are robust, exhibiting resistance to extremes of temperature, and desiccation (Williams et al., 1989). However, they can be lost from the soil *via* leaching (Dabiré and Mateille, 2004; Cetintas and Dickson, 2005; Luc et al., 2010). Trudgill et al. (2000) reported attachment of *P. penetrans* was favored by decreasing coarse sand and increasing clay content in Senegal but decreasing clay and organic matter content in Burkina Faso, with no such observable environmental effects on populations from Ecuador. However, due to reduced porosity and the ability of spores to bind to colloids, the presence of clay has been shown to improve retention of spores in the upper soil profile (Dabiré et al., 2007). The vast majority of *Pasteuria* spp. ecology research to date has examined a single species, *Pasteuria penetrans*. This is with good reason as *P. penetrans* is a parasite of the most significantly damaging PPNs globally, the tropical apomictic root knot nematodes (RKN, *Meloidogyne* spp.) (Davies et al., 2011; Jones et al., 2013). Some variation between populations of this species is observable as noted above with regard to the impact of soil clay content on retention of endospores (Trudgill et al., 2000). Other factors are more consistent, for example rate of development of *P. penetrans* has been shown to increase linearly between 18° and 27°C in multiple studies (Giannakou et al., 1997; Serracin et al., 1997; Lopes et al., 2018). However, it is possible that focus on this species obscures a greater diversity of endospore properties and environmental interactions within the genus given the high diversity and global distribution of *Pasteuria* species (Chen and Dickson, 1998). For example, despite the apparent negative impact of leaching on *P. penetrans* endospore retention, Costa et al. (2006) were readily able to recover *Pasteuria* spp. directly from sandy soils in temperate dunes. *Pasteuria hartismeri*, a parasite of temperate RKN species (Bishop et al., 2007), may be expected to be better suited to the lower temperatures within its distribution. Understanding the diversity, or lack thereof, of the relationships between *Pasteuria* spp. and environmental characteristics may therefore be critical to its effective deployment as a BCA.

Morphological diversity within *Pasteuria* spp. endospores manifests in differences in the shapes of the sporangial walls resultant from the arrangement of parasporal fibers (Starr and Sayre, 1988). For example, *P. ramosa*, which parasitizes the water flea (*Daphnia magna*) forms a characteristic “tear-drop” shape, whereas *P. penetrans* possesses a distinctive “flying saucer” or “fried egg” shape resulting from an inferiorly collapsed sporangia forming a concave cup like surface on the underside of the endospore (Starr and Sayre, 1988). The classical “flying saucer” shape of *P. penetrans* is common to a number of species which parasitize PPNs including *P. hartismeri* (Bishop et al., 2007) and *P. nishizawae*, which parasitizes the soybean cyst nematode (*H. glycines*) (Noel et al., 2005). *Pasteuria thornei*, which parasitizes semi-endoparasitic root lesion nematodes (*Pratylenchus* spp.) maintains a rigid rhomboidal structure (Starr and Sayre, 1988). Morphological characterization is, however, insufficient to identify most *Pasteuria* species and is therefore inadequate to predict interactions with the host. Molecular detection and characterization of *Pasteuria* spp. has been demonstrated using a variety of housekeeping genes, such as *GyrB* and *SigE*, (Schmidt et al., 2004; Nong et al., 2007; Mauchline et al., 2010; Mauchline et al., 2011) and the 16S rRNA gene (Duan et al., 2003; Atibalentja et al., 2004; Rao et al., 2012). Detection of single *Pasteuria* endospores was reported by Mauchline et al. (2010) using a commercial enzymatic digest followed by multiple strand amplification. PCR based detection has also been tested both in planta (Atibalentja et al., 2000; Schmidt et al., 2004; Rao et al., 2012) and in soils known to contain *Pasteuria* spp. (Duan et al., 2003). While these methods allow the molecular characterization of *Pasteuria* spp. populations and have provided the beginnings of a species 16S gene reference database, they are limited in both scale and depth. PCR and Sanger sequencing of individual samples with *Pasteuria* specific primers allows for few samples to be processed at any one time and, in complex populations, this method is unlikely to provide adequate sequencing depth to accurately characterize population diversity. We have developed a high throughput, high resolution method of determining *Pasteuria* spp. population structure which builds on existing molecular detection methods to increase their scale and depth. Here we demonstrate the utility of this method to assess the distribution and variation in *Pasteuria* spp. community structure in a range of Scottish soils and how such variation relates to the physical properties of the soils they inhabit. Further, we demonstrate that this approach can be combined with recent advances in high throughput nematode community profiling (Porazinska et al., 2009; Porazinska et al., 2010; Morise et al., 2012; Porazinska et al., 2012; Treonis et al., 2018; Waeyenberge et al., 2019) to assess the relationships that *Pasteuria* spp. may have on nematode community structure in agricultural soils. We describe significant species level variation in relationships with soil properties. The methodology described herein provides a framework which may be used or improved to study *Pasteuria* spp. ecology at scale and depth.

## Materials and Methods

### Environmental DNA Samples

#### East of Scotland Farm Network

Nematode communities were extracted from soils in the 2014 re-sampling of the East of Scotland Farm Network (ESFN) (Hawes et al., 2010) (n = 560) using a modified Baermann funnel extraction method as described by Brown and Boag (1988) with 200 g of soil used for each extraction (Wiesel et al., 2015). Extracted nematodes were lyophilized before DNA extraction with a PureLink® Pro 96 well Genomic DNA Purification Kit (Thermo Fisher Scientific) according to the manufacturer's instructions. The concentration of extracted DNA from ESFN samples was not measured.

#### National Soil Inventory Scotland 2

The National Soil Inventory Survey 2 (NSIS2) comprises 406 soil samples collected at 195 sites between 2007 and 2010 (Lilly et al., 2011). Pits, one meter in depth, were dug at 20 km grid intervals across the Scottish Isles and mainland and soil samples were collected for each horizon. Satellite soil samples were taken at fixed distances from selected main sample pits. DNA was extracted from 0.25 g of each soil sample, quantified using Quant-iT PicoGreen dsDNA assay kit (ThermoFisher), and stored at −80°C. Extensive soil and environmental metadata was collected corresponding to each site (Lilly et al., 2011). One hundred thirty archived NSIS2 DNA samples were removed from storage at −80°C for inclusion in our analysis. These samples were selected based on the following criteria: only upper soil horizons were included; satellite samples, 4–16 m from the main sample pit, were selected where available allowing assessment of variation at a site; and 11 NSIS2 sample sites, indicated to contain *Pasteuria*-like sequences based on shallow non-specific 16S rRNA gene sequence analysis of the NSIS2 dataset (Freitag, unpublished data), were included in order to characterize populations at these sites. Each NSIS2 sample was diluted to a concentration of 10 ng µl^−1^ in high performance liquid chromatography (HPLC) grade water before amplification.

### *Pasteuria* 16S rRNA Gene Copy Number Detection Limits

To determine an approximation of the sensitivity of our *Pasteuria* spp. PCRs, serial dilutions of *Pasteuria* spp. plasmids carrying cloned 16S rRNA gene PCR product were prepared. 16S rRNA gene sequences were amplified from *P. penetrans* genomic DNA and ESFN DNA. 16S rRNA gene product was amplified using primers 39F and 1166R (**Table 1**) as previously described (Mauchline et al., 2010) and ligated into pGEM-T easy plasmid (Promega) following the manufacturers protocol. Plasmids were used to transform electrocompetent DH5α cells from which individual clone colonies were cultured and plasmid extracted using the QIAprep spin kit (QIAGEN). The sequence of clone plasmids was confirmed with Sanger sequencing using generic M13F and M13R primers. Plasmids were quantified using a Qubit dsDNA HS Assay Kit (Thermo Fisher Scientific). The copy number of plasmid template µl^−1^ was calculated from the combined length of vector and insert and the concentration of each plasmid suspension (ng μl^−1^) using the formula:

$$\mathrm{copy}\;\mathrm{number}\;=\;\frac{\mathrm{dsDNA}\;\mathrm{mass}\;\left(\mathrm{ng}\right)\;\times \;(6.022\times 10^{23})}{\mathrm{size}\;\mathrm{of}\;\mathrm{plasmid}\;\left(\mathrm{bp}\right)\times \left(650\times 10^{9}\right)}$$

**Table 1:** Primers used in this study.

Primer	Sequence (5’-3’)	Target gene	Reference
39F	GCGGCGTGCCTAATACA	16S rRNA	Atibalentja et al., 2000
1166R	CGCCGGCTGTCTCTCCAA	16S rRNA	Duan et al., 2003
PAS776F	CAGCATCTTTGTGCCGAAGG	16S rRNA	This Study
NF1	GGTGGTGCATGGCCGTTCTTAGTT	18S rRNA	Mullin et al., 2003
18Sr2b	TACAAAGGGCAGGGACGTAAT	18S rRNA	Mullin et al., 2003

To assess approximate gene copy number detection limits dilutions were prepared of *P. penetrans* and *P. hartismeri* 16S rRNA gene plasmid stocks to a concentration which was calculated to corresponded to ~10 million copies of each plasmid. This stock was then used to produce six replicate serial dilution series of each plasmid from an initial estimate of 10 million copies to a theoretical single copy of target sequence µl^−1^ of plasmid suspension. One µl of each plasmid dilution series was included as template in PCR reactions alongside NSIS2 and ESFN samples in *Pasteuria* PCR reactions as below and included in the final PCR product pool prepared for sequencing.

### Amplification Strategy

#### *Pasteuria* spp. Primer Design

**Figure 1:**
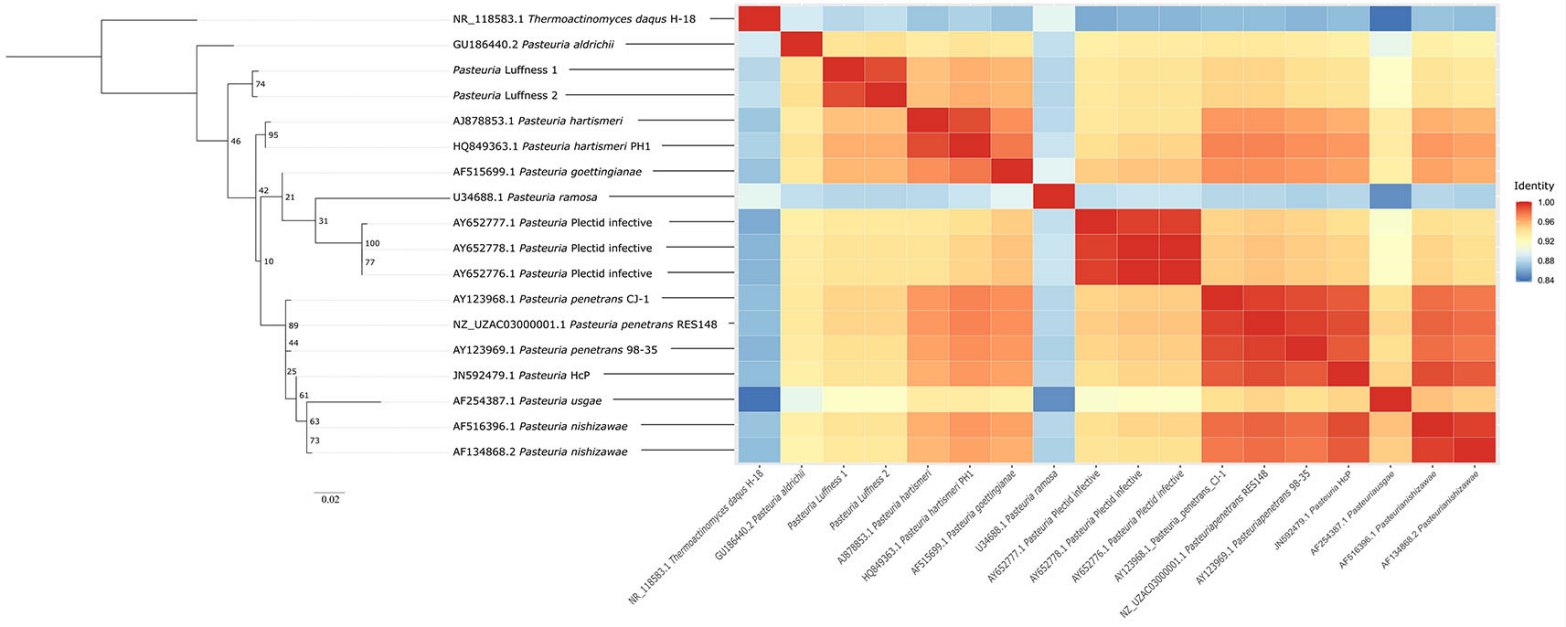
Maximum likelihood phylogeny and pairwise sequence identity heatmap of *Pasteuria* spp. taxonomic reference sequences used in primer design and *Pasteuria* spp. ZOTU taxonomic assignment.

All primer sequences used in this study are presented in **Table 1**. Primers for the specific amplification of the *Pasteuria* 16S rRNA gene were designed based on published 16S rRNA gene data. All *Pasteuria* spp. 16S rRNA gene sequences (n = 272) were downloaded from the non-redundant nucleotide archive. These sequences were aligned using MAFFT (v7.407) (Katoh and Standley, 2013) and trimmed using a python script (this study) to those sequences containing 39F and 1166R primer sites allowing for a mismatch of up to 3 nt (n = 54) which excluded shorter sequence fragments. The remaining sequences were used as input in Primer-BLAST (Ye et al., 2012) as target sequences with primer 1166R as the input reverse primer and a maximum PCR product size of 500 bp. Primer-BLAST was then used to predict non-target amplification of candidate forward primer-1166R pairs vs the non-redundant nucleotide archive allowing up to six mismatches in total within the primer pair (Ye et al., 2012). The primer pair PAS776F (this study) and 1166R (Duan et al., 2003), generating a 333 bp fragment showed no *in silico* predicted non-target amplification. The reference set was again trimmed to the region between primers PAS776F and 1166R using python (this study) and aligned using MAFFT (v7.407) (Katoh and Standley, 2013) with the addition of 16S rRNA gene sequences for *P. ramosa*, which does not carry the primer binding sites, and *Thermoactinomyces daqus*, a related Firmicute (Ludwig et al., 2009). Using this alignment, it was determined that all previously described species were distinguishable by at least two bp in the target region. Identical sequences, and sequences unassigned to a particular species or host, were removed from the reference set leaving 16 representative sequences: *T. daqus*, (n = 1), *P. ramosa* (n = 1), *P. penetrans* (n = 3), *P. hartismeri* (n = 2), *P. nishizawae* (n = 2), *P. goettingianae* (n = 1), *P. usgae* (n = 1), *P. aldrichii* (n = 1), Plectid infective *Pasteuria* spp. isolates (n = 3), and *Pasteuria* HcP (n = 1). In addition, *Pasteuria*-like sequences aligning with 97% similarity to *P. hartismeri* were added which we have called here *Pasteuria* Luffness (n = 2) for the location of their initial recovery bringing the total reference set to 18 sequences. These sequences were used as the reference database for taxonomic assignment of *Pasteuria* spp. amplicons. The pairwise percentage identity of reference *Pasteuria* spp. sequences in the aligned region of the 16S rRNA gene ranged from a low of 84.7% (*P. ramosa* vs *P. usgae*) to a high of 99.3% (*P. penetrans* to *Pasteuria* HcP) with an average of 95% identity between all reference sequences. Maximum likelihood phylogeny was inferred from aligned reference sequences using IQ-TREE (v1.6.9) (Nguyen et al., 2014) with bootstrapping (n = 100), and graphically represented in FigTree (v1.4.3) (Rambaut, 2012) (**Figure 1**). The pairwise similarity of reference sequences was plotted as a heatmap (**Figure 1**) using ggplot2 (Wickham, 2009).

#### Primer Barcode Design

A pairwise in-line primer barcoding strategy was devised to index PCR products. Barcode sequences (6 nt) were designed based on a Levenshtein distance of three using the EditTag python package (Faircloth and Glenn, 2012). These were trimmed to 32 tags (1024 pairwise combinations) based on minimizing penalty scores generated using the same software. Two adenine bases were appended to the 5' end of each barcode to account for base loss at the beginning of a sequencing read. Pairwise combinations of barcoded primers were achieved by diluting the stocks from 100 µM to 0.6 µM and then adding 2.5 µl of each primer to a respective reaction, each individual primer in 32 unique reactions.

#### Amplification

PCR amplification of *Pasteuria* spp. 16S rRNA gene sequence was carried out using a semi-nested approach using 1 µl of ESFN, NSIS2, or plasmid dilution series DNA as template. Large fragment (1110 bp) 16S rRNA gene products were amplified using primers 39F (Atibalentja et al., 2000) and 1166R. This product was then diluted 1 in 10 and used as template for short, barcoded inner nest PCR that used primers PAS776F and 1166R. Outer nest PCR conditions were 94°C for 5 minutes, followed by 30 cycles of 94°C for 1 minute, 60°C for 1 minute, and 72°C for 1 minute, with a final extension at 72°C for 10 minutes. PCR conditions for *Pasteuria* inner nest reactions included an initial denaturation of 94°C for 5 minutes, followed by 15 cycles of 94°C for 15 seconds, 70°C for 20 seconds, and 72°C for 20 seconds, with a final extension at 72°C for 1 minute.

18S rRNA gene sequences were amplified from ESFN DNA samples using primers NF1 and 18Sr2b (Mullin et al., 2003; Porazinska et al., 2009). PCR conditions were 94°C for 5 minute, followed by 40 cycles of 94°C for 30 seconds, 58°C for 30 seconds, and 72°C for 1 minute, with a final extension at 72°C for 10 minute.

All reactions (20 µl) were carried out with Q5 High-Fidelity DNA Polymerase (NEB) with 1 µl of DNA as template. A high-fidelity polymerase with 3–5′ exonuclease activity was used in all PCR reactions to minimize artificial sequence variation generated by PCR errors.

### Library Preparation From PCR Products

Each short PCR product (4 μl) was electrophoresed on a 2% agarose gel. Gels were visualized for bands of the expected size and each band was assigned a score from 1 to 4 based on its relative brightness: 1, not visible; 2, barely visible; 3, clearly visible; and 4, very bright. This scoring was used to approximately normalize PCR product concentrations, to avoid overrepresentation of concentrated reactions, as follows: 10 μl (scoring 1 or 2); 5 μl (scoring 3); and 1 μl (scoring 4) of products added to the final product pool. All products from PAS776 and 1166R PCRs of NSIS2, ESFN, and control DNA samples were pooled; all products from NF1 and 18Sr2b PCRs of ESFN DNA were pooled separately. Once pooled, products were concentrated by overnight incubation at −80°C with the addition of 1 volume of isopropanol, 0.2 volumes of 3M sodium acetate, and 20 ng of glycogen, followed by centrifugation at 10,600 g for 15 minutes at 4°C to pellet DNA. The supernatant was discarded, the pellet washed in 70% ice cold ethanol and allowed to air dry. Dried pellets were re-suspended in 105 μl of HPLC water. Pooled, concentrated PCR products were subjected to size selection using the MagJET NGS Cleanup and Size Selection Kit (Thermo Fisher Scientific). This size selection was carried out to exclude primers and, in the case of nested *Pasteuria* spp. 16s rRNA gene PCRs, to exclude outer nest PCR products. Binding buffer (400 µl) was used with both initial and final bead binding incubations which were extended to 15 minute, and intermediate binding was kept at 2 minutes to maximize product recovery. PCR product pools were combined and prepared for sequencing using the TruSeq PCR-Free Library Preparation Kit (Illumina), omitting shearing. The TruSeq PCR-Free Library Preparation Kit was selected to minimize sequence errors introduced by amplification. Sequencing was carried out at Edinburgh Genomics on Illumina’s MiSeq platform with 300 base paired end reads.

### Read Trimming and Overlap Merging

Initial sequence quality was assessed using FastQC v0.11.3 (Andrews, 2010). Trimmomatic v0.33 (Bolger et al., 2014) was used to trim reads with a minimum Phred quality score of 22 and a minimum length of 150 bp. Trimmomatic parameters were determined by incremental reduction in quality score cut-offs until >75% of all paired reads could be merged. PEAR v0.9.6 (Zhang et al., 2014) was used to merge quality trimmed paired end reads. Primer sequences were used to separate merged read pairs into amplification target groups, using primer regular expression matching with an acceptable ambiguity of 1 nt, and their orientation was then corrected using Python (this study). VSEARCH v2.1.1 (Rognes et al., 2016) was used to filter merged read pairs for a maximum number of expected base calling errors of less than 2 (Edgar and Flyvbjerg, 2015). Merged read pairs were binned in respective sample FASTQ files based on in-line primer barcode sequences, trimmed to exclude sequence up to and including primer sequences, to confine sequences to variable regions, then renamed to meet downstream requirements using Python (this study).

UNOISE3 from USEARCH v.10.0.240 (Edgar, 2013; Edgar, 2016) was used to generate zero radius operational taxonomic units (ZOTUs). ZOTU is a term specific to analysis with UNOISE referring to operational taxonomic units (OTUs) which are generated by an error correction algorithm as opposed to a sequence similarity clustering algorithm (Edgar, 2016). Raw merged read pairs were mapped back to ZOTUs using the otutab command in USEARCH.

### Taxonomic Assignment

Taxonomy was assigned to ZOTUs using UCLUST (Edgar, 2010) *via* assign-taxonomy.py in QIIME v1.91 (Caporaso et al., 2010) to cluster ZOTU sequences iteratively between 100% and 90% identity to our curated *Pasteuria* spp. reference database for 16S rRNA gene products and to the SILVA-132 database (Quast et al., 2012) for the 18S rRNA gene products. All reference sequences were trimmed to the regions between the primer sequences allowing for a maximum mismatch of 1nt in each primer sequence. Iterative best hit assignment was used as each sequence being assigned taxonomy was a single representative of a putatively real biological sequence. The percentage match of each taxonomic assignment was appended to ZOTU identifiers as an integer between 0.9 and 1.0 (Quast et al., 2012). Iterative taxonomy tables were combined into a single table containing the best available match for each ZOTU sequence using Python (this study). Thereafter, ZOTU tables were combined with sample metadata and best taxonomic assignments using R scripts (this study).

### ZOTU Table Quality Control

ZOTU tables with metadata were filtered for total ZOTU abundance (> 10) across the entire dataset to eliminate uninformative, low abundance ZOTUs. 18S rRNA gene products were also filtered for unwanted taxa to exclude ZOTUs outside of Nematoda. The number of reads generated for each sample was compared against the PCR band score to determine that observed amplification matched with the abundance of assembled products. Comparisons of these two independent estimates of PCR product mass were used to assess the likelihood of errors in the barcode sorting pipeline, pipetting of barcoded primers, sample storage, and cross contamination of barcode primer stocks. Samples which showed sustained mismatch between band scoring and merged paired read counts were removed.

### Ordination and Statistical Analysis

*Pasteuria* spp. communities in the NSIS2 samples and nematode communities in the ESFN samples were ordinated using non-metric multidimensional scaling (NMDS) using Vegan (Dixon, 2003). ESFN samples which did not produce visible metazoan PCR product were excluded as uninformative. Soil metadata and ZOTU abundance were then fitted to these ordinations using Vegan’s envfit. P values were adjusted for the false discovery rate using the Benjamini-Hochberg correction method (Benjamini and Hochberg, 1995).

### *Pasteuria*-Nematode ZOTU Interactions

*Pasteuria* and nematode ZOTU correlation was computed as the Spearman's rank correlation between each pairwise combination of ZOTUs. This was computed in R using a slightly modified version of a script described by Williams et al. (2014) designed to determine microbial co-occurrence patterns. P values were adjusted for the false discovery rate using the Benjamini-Hochberg correction method (Benjamini and Hochberg, 1995).

### Nematode Recovery

Recovery of nematodes from ESFN soils which returned a large 18S rRNA gene fragment PCR product was carried out to attempt to confirm the presence of *Pasteuria* endospores in soils which returned *Pasteuria*-like PCR products. This was done using a high density sucrose flotation method designed to optimize recovery of dense endospore filled nematodes (Hewlett et al., 1997). Two hundred grams of ESFN soils were suspended in 600 ml of tap water and the slurry was shaken vigorously for a period of 2 minutes then passed through a bank of sieves (250, 90, 25 μm). The retentate from the 25 μm sieve was re-suspended in approximately 30 ml of sterile distilled water and this was spun at 420 g for 5 minutes to pellet nematodes. The supernatant was discarded, and the pellet suspended in approximately 30 ml of sterile sucrose solution with an approximate density of 1.28 g ml^−1^. This was spun again at 420 g for 1 minute and the supernatant passed through a 25 μm sieve. The retentate from this sieve was re-suspended in approximately 20 ml of SDW and 4 x 5 ml observed in a counting dish for the presence of endospores with an inverted microscope (Hund Wilovert^®^) at 50 and 200X magnification.

### Immunofluorescence

Immunofluorescent labeling of *P. penetrans* spores on live nematodes recovered from soils was carried out using a previously described polyclonal antibody (Costa et al., 2006). A 1:1000 dilution of antibody was added to an equal volume of sterile distilled water containing the recovered nematode. This was left for 1 hour at room temperature or at 4°C overnight before being washed by successive transfer of individual nematodes to 5 ml of sterile distilled water. The resultant solution was then incubated with Goat anti-rabbit IgG bound to red fluorescent dye CyC at 4°C overnight. This was washed of unbound antibody as before and viewed under RFP fluorescent microscopy at 200 and 1,000x magnification with a Zeiss Axiosop microscope.

## Results

### PCR

Of 560 ESFN nematode DNA samples 266 (47.5%) and 122 (21.8%) score of 2 or higher with metazoan and *Pasteuria* spp. primers, respectively. Considering ESFN samples which did not amplify as failed DNA extractions or PCR reactions, 45.9% of successful ESFN samples returned *Pasteuria* spp. PCR product. Of 144 NSIS2 soil DNA samples, 56 (38.9%) score 2 or higher using *Pasteuria* spp. PCR primers.

### Size Selection

Extension of initial and final bead binding incubations, to the exclusion of primers and shorter products, and reduction of the intermediate binding, to the exclusion of larger PCR products, increased the efficiency of PCR product recovery from just 6% to 35% with an approximate input of 5 μg.

### Sequence Pipeline

Out of a total of 8,945,443 raw paired end reads, 77.8% remained after trimming; of these 98.7% were merged. Merged read pairs produced 1,181,554 *Pasteuria* spp. 16S rRNA gene sequences; 2,100,892 metazoan 18S rRNA gene sequences; and 183,079 matching no primer sequence. Ninety-nine percent of the metazoan and 92% of the *Pasteuria* spp. merged read pairs remained after expected error and barcode sorting. Four percent of the *Pasteuria* spp. and 16% of the metazoan merged read pairs were unique sequences.

### Nematode Primer Specificity

Nematode ZOTUs account for 72% of all ZOTUs and 85% of all merged read pairs within the ESFN 18S rRNA gene sample set. Annelid worms comprised 12% of the assembled read pairs and 4% of ZOTUs in this set. Fungi, Oomycetes, and Alveolata each comprised 1% of remaining assembled read pairs.

### *Pasteuria* Detection Limits

**Figure 2:**
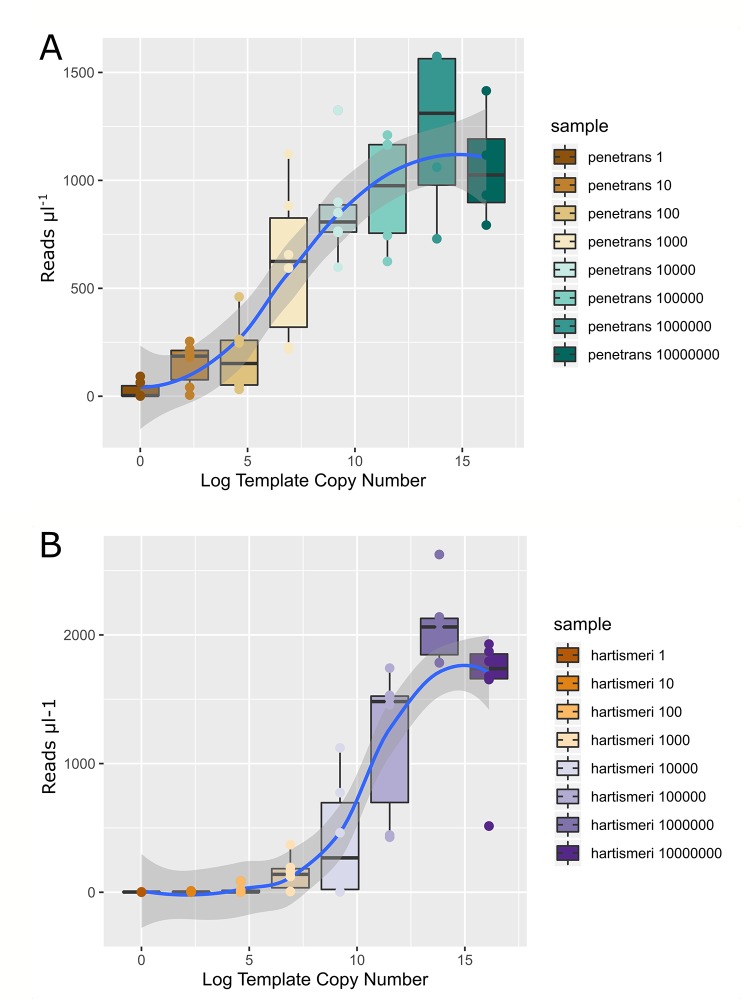
Boxplots of *Pasteuria penetrans* **(A)** and *P. hartismeri* **(B)** 16S rRNA gene template copy number vs the merged paired read copy number ul^−1^ of product added to the final sequencing pool. Spearman's rank correlation is given as Rho and p. Loess curve of best fit is given as the blue trend line with dark grey shading representing uncertainty in this fit with a 95% confidence interval. Template copy number, representing the number of plasmids in each PCR reaction carrying the target gene, was log transformed as each input copy number was an order of magnitude greater or less than the next smallest or largest.

Amplification and agarose gel electrophoresis demonstrated a typical detection limit of approximately 1000 target gene copies. Sequencing results largely reflected this with consistent detection of *P. penetrans* and *P. hartismeri* target sequences at 1,000 target copies and above (**Figure 2**). Reads obtained from sequencing µl−^1^ of PCR product do not increase beyond 1,000,000 target gene copies in the PCR reaction.

### Diversity and Distribution

**Figure 3:**
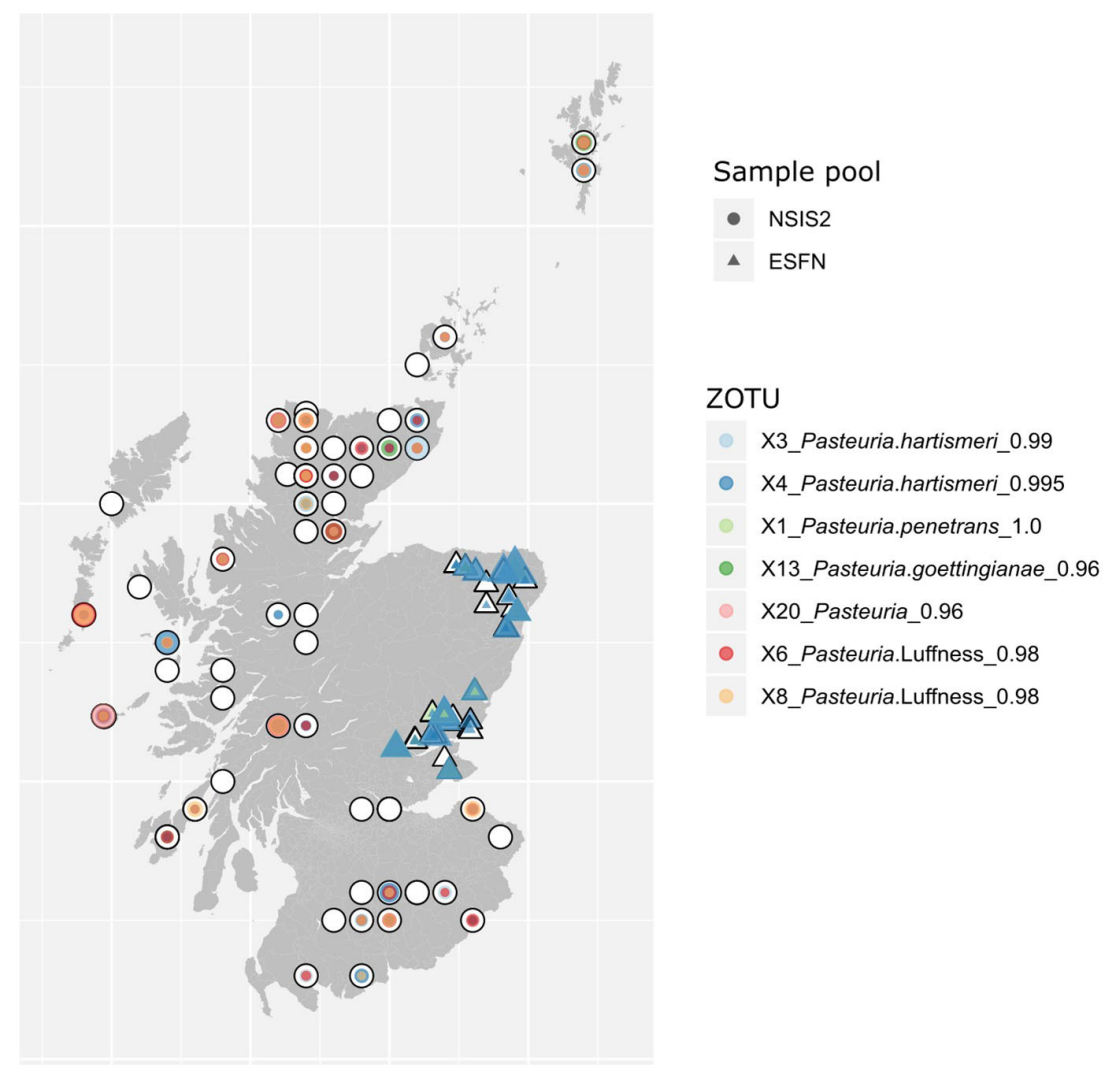
Map of distribution of the most abundant *Pasteuria* spp. ZOTUs across both NSIS2 (circles) and ESFN (triangles) datasets. Plot points are colored by ZOTU and sized as a function of the total number of merged read pairs recovered for that ZOTU µl^−1^ of product added to the final pool from the corresponding sample.

Soil samples from the NSIS2 which contained *Pasteuria* spp. were broadly distributed with no sequence or ZOTU variant bearing a significant relationship to easting or northing (**Figure 3**). The most common and widely distributed sequence variants recovered matched most closely to reference sequences for *Pasteuria hartismeri* and a sequence recovered at a farm near Edinburgh here referred to as “*Pasteuria* Luffness”. ESFN samples were similarly dominated by these two sequences at most sites.

#### *Pasteuria* ZOTUs vs Soil Properties

**Figure 4:**
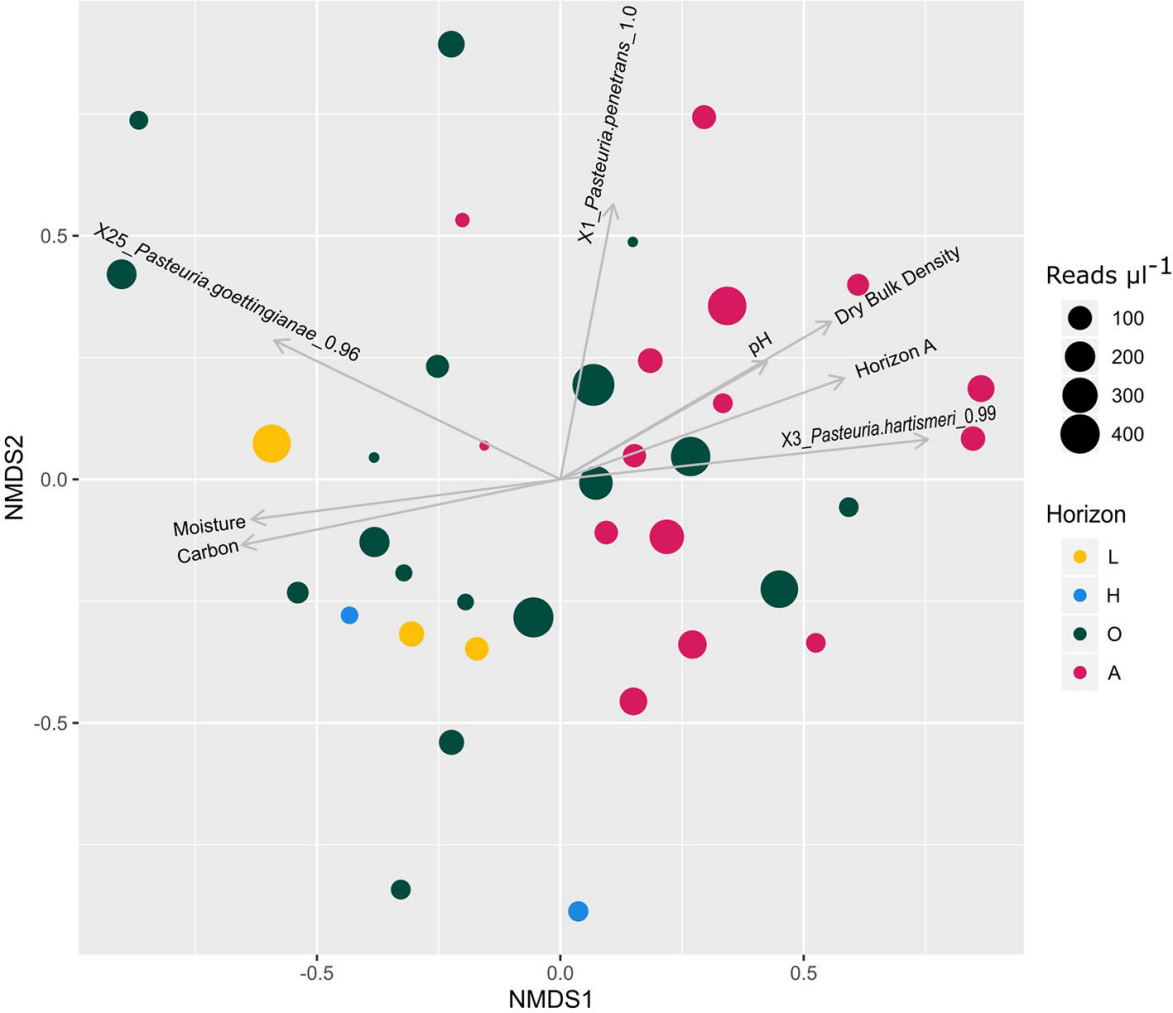
NDMS ordination plot of NSIS2 *Pasteuria* spp. community composition. Points are colored by soil horizon where L = litter; H = humus; O = peaty material formed under wet, anaerobic conditions; and A = mineral horizon formed at or near the surface showing accumulation and incorporation of organic matter. Points are sized by the total number of merged read pairs µl^−1^ of PCR product added to the final pool from the corresponding sample. Stress = 0.245235.

**Figure 4** shows the plotted NMDS ordination and associated metadata and species fits. Statistically significant relationships were observed between *Pasteuria* ZOTU sequence variants and near surface mineral horizon A, soil carbon, dry bulk density, pH, and field moisture (**Table 2**).

**Table 2:** Environmental variables with a statistically significant relationship to *Pasteuria* spp. community ordination before or after Benjamini-Hochberg correction.

	NMDS1	NMDS2	r	P	BH adjusted P
Horizon: A	0.583	0.208	0.38	0.001	0.01
Bulk Density	0.556	0.324	0.41	0.001	0.01
Carbon	−0.654	−0.135	0.45	0.001	0.01
Moisture	−0.634	−0.082	0.41	0.001	0.01
pH	0.425	0.242	0.24	0.006	0.05
Vegetation ES1C	−0.334	0.314	0.21	0.019	0.13
Clay	0.385	0.210	0.19	0.022	0.13
Major Soil Group: Peat	−0.438	0.005	0.19	0.033	0.17
Horizon: O	−0.388	0.041	0.15	0.053	0.24

Major soil group peat, vegetation code ES1C (terminal phase blanket bog), clay content, and soil horizon O were significant factors pre-Benjamini-Hochberg correction, however, these drop from significance post-correction. Other soil horizons, major soil groups, and vegetation codes lacked statistically significant effects, both before and after Benjamini-Hochberg correction, on *Pasteuria* community composition.

NSIS2 *Pasteuria* spp. soil samples do not cluster into clear independent groups in the NMDS analysis. This suggests that the relationship between the above factors and the abundance of recovered ZOTUs is not binary but scalar. For example, ZOTU X3 assigned to *P. hartismeri* with 99% identity is more abundant in samples with less carbon, a higher pH, and a lower moisture content. These variables have strong pairwise Spearman's rank correlations (**Table 3**) and likely summarize the general properties of the soil horizon A in contrast to organic horizons L, H, and O. ZOTU X25, assigned with 96% identity to *Pasteuria goettingianae* has the inverse relationship with these variables, while X1, which is most similar to *P. penetrans* in the amplified region, shows no strong preference. However, each ZOTU is also present in organic and mineral soils/horizons at lower abundance.

**Table 3:** Spearman’s rank correlation of soil properties with statistically significant correlation to *Pasteuria* spp. community composition.

	Horizon A	Dry Bulk Density	Carbon	pH	Field Moisture
Horizon A	1	0.84	−0.85	0.84	−0.83
Dry Bulk Density	0.84	1	−0.78	0.78	−0.90
pH	0.84	0.78	−0.66	1	−0.75
Field Moisture	−0.83	−0.90	0.85	−0.75	1
Carbon	−0.85	−0.78	1	−0.66	0.85

### *Pasteuria*-Nematode Interactions

#### ESFN Nematode Communities

**Figure 5:**
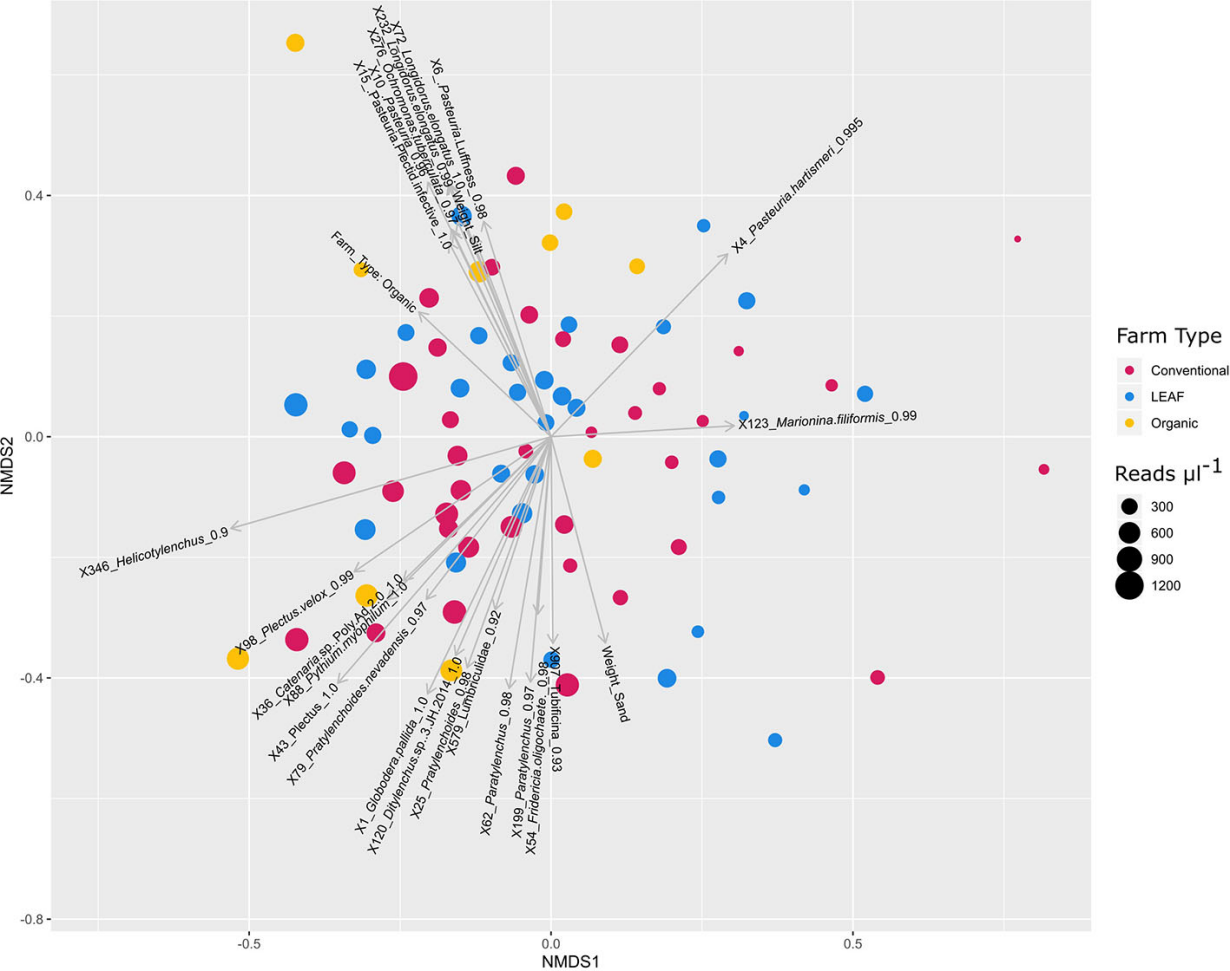
NMDS ordination of ESFN nematode community ZOTUs. Each point represents a nematode community. Samples are colored by farm type and sized by the total abundance of nematode merged read pairs recovered µl^−1^ of PCR product added to the final pool. Arrows represent the statistically significant fits of Nematode ZOTUs, *Pasteuria* ZOTUs, non-nematode metazoan ZOTUs, and environmental factors where the length of the arrow reflects the effect size. For ease of visualization the number of significant nematode ZOTUs plotted has been reduced. Stress = 0.2482174.

Organic farming methods show a statistically significant relationship (P = 0.035) with nematode community structure pre-Benjamini-Hochberg correction although LEAF and conventional farms do not (**Figure 5**). No soil environmental factors tested in the ESFN dataset remained significant (P < 0.05) following Benjamini-Hochberg correction. However, the weight of sand (P = 0.009) and silt (P = 0.013) in each sample appeared to significantly influence the nematode community before correction.

Several ZOTUs bearing sequence similarity to PNN genera drive diversity in nematode community structure (**Figure 5**). *Pasteuria hartismeri*-like ZOTU X4 displays a negative fit to almost all statistically significant nematode ZOTU sequences. *Pasteuria* spp. ZOTU sequences X15 assigned to a *Pasteuria* spp. infective of bacterial feeding Plectidae, correlates with organic farms, silt weight, and *Longidorus elongatus* (Tzean and Estey, 1981).

Direct PCN J2 counts were available for 74 of the ESFN samples taken from a potato variety and nematicide incorporation trial conducted in two fields at a single farm. Direct J2 counts and ZOTUs assigned to *Globodera* spp. showed statistically significant (P = 3.25e^−07^) positive correlation. However, this correlation was weak (R = 0.67).

#### ZOTU Spearman’s Rank Correlations

To test direct species to species interactions, rather than overall community interactions, the pairwise Spearman's rank correlation of all nematode and *Pasteuria* spp. ZOTUs was calculated. Several *Pasteuria* and nematode ZOTUs bear a statistically significant relationship (**Table 4**). However, the observed correlations are typically weak. *Pasteuria* spp. ZOTUs X1, X2, X3, and X4 display a significant negative correlation with metazoan ZOTUs assigned to *Paratylenchus* species. ZOTU X2, and X3 both assigned to *P. hartismeri* also displays a statistically significant negative correlation with *Heterodera* and *Meloidogyne* species.

**Table 4:** The top three most abundant *Pasteuria* spp. ZOTUs in the ESFN dataset and respective metazoan ZOTU Spearman’s rank correlations which were statistically significant after Benjamini-Hochberg correction.

ZOTU 1	ZOTU 2	rho	P	BH corrected P
X4_*Pasteuria.hartismeri*_0.995	X701_*Filenchus.discrepans*_1.0	0.25	7.7E-05	2.4E-03
X4_*Pasteuria.hartismeri*_0.995	X851_*Ditylenchus.dipsaci*_0.98	0.23	2.1E-04	5.5E-03
X4_*Pasteuria.hartismeri*_0.995	X867_*Longidorus*_0.96	0.23	2.1E-04	5.6E-03
X4_*Pasteuria.hartismeri*_0.995	X845_*Pratylenchoides*_0.98	0.23	2.2E-04	5.8E-03
X4_*Pasteuria.hartismeri*_0.995	X58_*Diphterophora*_0.98	0.23	2.5E-04	6.5E-03
X4_*Pasteuria.hartismeri*_0.995	X70_*Diphterophora*_0.98	0.21	7.2E-04	1.5E-02
X4_*Pasteuria.hartismeri*_0.995	X72_*Longidorus.elongatus*_1.0	0.21	8.6E-04	1.8E-02
X4_*Pasteuria.hartismeri*_0.995	X876_Ecumenicus_0.98	0.20	1.8E-03	3.2E-02
X4_*Pasteuria.hartismeri*_0.995	X784_Cephalobidae_0.94	−0.20	1.2E-03	2.3E-02
X4_*Pasteuria.hartismeri*_0.995	X65_*Heterocephalobus.elongatus*_1.0	−0.21	6.6E-04	1.4E-02
X4_*Pasteuria.hartismeri*_0.995	X752_*Heterodera*_0.97	−0.23	3.3E-04	8.2E-03
X4_*Pasteuria.hartismeri*_0.995	X706_Tylenchidae_0.93	−0.23	2.5E-04	6.4E-03
X4_*Pasteuria.hartismeri*_0.995	X846_Tylenchoidea_0.93	−0.24	1.1E-04	3.1E-03
X4_*Pasteuria.hartismeri*_0.995	X657_Tylenchidae_0.91	−0.26	3.2E-05	1.1E-03
X4_*Pasteuria.hartismeri*_0.995	X800_*Eucephalobus*.cf.oxyuroides.JH.2004_0.97	−0.26	2.3E-05	8.5E-04
X4_*Pasteuria.hartismeri*_0.995	X55_*Thonus*.sp.JH.2004_1.0	−0.27	1.7E-05	6.6E-04
X4_*Pasteuria.hartismeri*_0.995	X641_Plectidae_0.92	−0.28	7.7E-06	3.2E-04
X4_*Pasteuria.hartismeri*_0.995	X5_*Meloidogyne*_1.0	−0.29	4.7E-06	2.1E-04
X4_*Pasteuria.hartismeri*_0.995	X68_*Eucephalobus*.cf.oxyuroides.JH.2004_0.995	−0.32	2.2E-07	1.3E-05
X4_*Pasteuria.hartismeri*_0.995	X56_*Aphelenchoides*.sp.JH.2004_0.97	−0.32	1.8E-07	1.1E-05
X4_*Pasteuria.hartismeri*_0.995	X75_Tylenchoidea_0.95	−0.35	9.9E-09	7.4E-07
X4_*Pasteuria.hartismeri*_0.995	X62_*Paratylenchus*_0.98	−0.37	1.8E-09	1.5E-07
X3_*Pasteuria.hartismeri*_0.99	X845_*Pratylenchoides*_0.98	0.31	4.0E-07	2.3E-05
X3_*Pasteuria.hartismeri*_0.99	X851_*Ditylenchus.dipsaci*_0.98	0.31	4.8E-07	2.6E-05
X3_*Pasteuria.hartismeri*_0.99	X701_*Filenchus.discrepans*_1.0	0.26	4.4E-05	1.5E-03
X3_*Pasteuria.hartismeri*_0.99	X867_*Longidorus*_0.96	0.24	9.7E-05	2.9E-03
X3_*Pasteuria.hartismeri*_0.99	X663_*Ditylenchus.dipsaci*_0.97	0.24	1.8E-04	4.9E-03
X3_*Pasteuria.hartismeri*_0.99	X70_*Diphterophora*_0.98	0.23	2.1E-04	5.6E-03
X3_*Pasteuria.hartismeri*_0.99	X798_*Ditylenchus.dipsaci*_0.995	0.22	4.0E-04	9.6E-03
X3_*Pasteuria.hartismeri*_0.99	X755_Tylenchidae_0.95	0.22	5.0E-04	1.2E-02
X3_*Pasteuria.hartismeri*_0.99	X549_*Neopsilenchus.magnidens*_0.97	0.21	1.0E-03	2.1E-02
X3_*Pasteuria.hartismeri*_0.99	X876_*Ecumenicus*_0.98	0.19	2.4E-03	4.0E-02
X3_*Pasteuria.hartismeri*_0.99	X784_Cephalobidae_0.94	−0.19	2.8E-03	4.5E-02
X3_*Pasteuria.hartismeri*_0.99	X706_Tylenchidae_0.93	−0.20	1.2E-03	2.3E-02
X3_*Pasteuria.hartismeri*_0.99	X5_*Meloidogyne*_1.0	−0.20	1.2E-03	2.3E-02
X3_*Pasteuria.hartismeri*_0.99	X56_*Aphelenchoides*.sp.JH.2004_0.97	−0.24	1.5E-04	4.1E-03
X3_*Pasteuria.hartismeri*_0.99	X55_*Thonus*.sp.JH.2004_1.0	−0.26	4.6E-05	1.5E-03
X3_*Pasteuria.hartismeri*_0.99	X641_Plectidae_0.92	−0.26	3.8E-05	1.3E-03
X3_*Pasteuria.hartismeri*_0.99	X657_Tylenchidae_0.91	−0.28	8.5E-06	3.5E-04
X3_*Pasteuria.hartismeri*_0.99	X68_*Eucephalobus*.cf.oxyuroides.JH.2004_0.995	−0.30	1.9E-06	9.3E-05
X3_*Pasteuria.hartismeri*_0.99	X75_Tylenchoidea_0.95	−0.30	1.5E-06	7.6E-05
X3_*Pasteuria.hartismeri*_0.99	X62_*Paratylenchus*_0.98	−0.32	3.2E-07	1.8E-05
X1_*Pasteuria.penetrans*_1.0	X887_Diphtherophorina_0.96	0.23	3.0E-04	7.5E-03
X1_*Pasteuria.penetrans*_1.0	X713_*Alaimus*.sp.PDL.2005_0.98	0.23	3.1E-04	7.7E-03
X1_*Pasteuria.penetrans*_1.0	X70_Diphterophora_0.98	0.21	6.5E-04	1.4E-02
X1_*Pasteuria.penetrans*_1.0	X615_*Nygolaimus*.cf.brachyuris.JH.2004_0.995	0.21	1.0E-03	2.1E-02
X1_*Pasteuria.penetrans*_1.0	X525_*Eumonhystera*.cf.filiformis.1.JH.2014_0.97	0.20	1.7E-03	3.1E-02
X1_*Pasteuria.penetrans*_1.0	X855_Aquatides_0.96	0.19	2.8E-03	4.5E-02
X1_*Pasteuria.penetrans*_1.0	X678_*Nygolaimus*.cf.brachyuris.JH.2004_0.99	0.19	3.0E-03	4.8E-02
X1_*Pasteuria.penetrans*_1.0	X56_*Aphelenchoides*.sp.JH.2004_0.97	−0.19	2.0E-03	3.5E-02
X1_*Pasteuria.penetrans*_1.0	X75_Tylenchoidea_0.95	−0.20	1.2E-03	2.3E-02
X1_*Pasteuria.penetrans*_1.0	X570_Plectus_0.94	−0.20	1.2E-03	2.3E-02
X1_*Pasteuria.penetrans*_1.0	X55_*Thonus*.sp.JH.2004_1.0	−0.22	5.8E-04	1.3E-02
X1_*Pasteuria.penetrans*_1.0	X657_Tylenchidae_0.91	−0.22	5.3E-04	1.2E-02
X1_*Pasteuria.penetrans*_1.0	X68_*Eucephalobus*.cf.oxyuroides.JH.2004_0.995	−0.23	2.9E-04	7.4E-03
X1_*Pasteuria.penetrans*_1.0	X641_Plectidae_0.92	−0.23	2.3E-04	5.9E-03
X1_*Pasteuria.penetrans*_1.0	X62_*Paratylenchus*_0.98	−0.24	1.8E-04	4.9E-03
X2_*Pasteuria*.Luffness_0.98	X92_*Xiphinema.pachtaicum*_1.0	0.26	2.4E-05	8.8E-04
X2_*Pasteuria*.Luffness_0.98	X867_*Longidorus*_0.96	0.24	1.3E-04	3.8E-03
X2_*Pasteuria*.Luffness_0.98	X734_*Longidorus*_0.97	0.21	7.2E-04	1.5E-02
X2_*Pasteuria*.Luffness_0.98	X701_*Filenchus.discrepans*_1.0	0.21	9.9E-04	2.0E-02
X2_*Pasteuria*.Luffness_0.98	X56_*Aphelenchoides*.sp.JH.2004_0.97	−0.19	2.6E-03	4.3E-02
X2_*Pasteuria*.Luffness_0.98	X657_Tylenchidae_0.91	−0.20	1.1E-03	2.2E-02
X2_*Pasteuria*.Luffness_0.98	X68_*Eucephalobus*.cf.oxyuroides.JH.2004_0.995	−0.21	6.8E-04	1.5E-02
X2_*Pasteuria*.Luffness_0.98	X62_*Paratylenchus*_0.98	−0.24	9.6E-05	2.9E-03
X2_*Pasteuria*.Luffness_0.98	X641_Plectidae_0.92	−0.25	7.4E-05	2.3E-03

#### Recovery of Endospore Encumbered Nematodes

**Figure 6:**
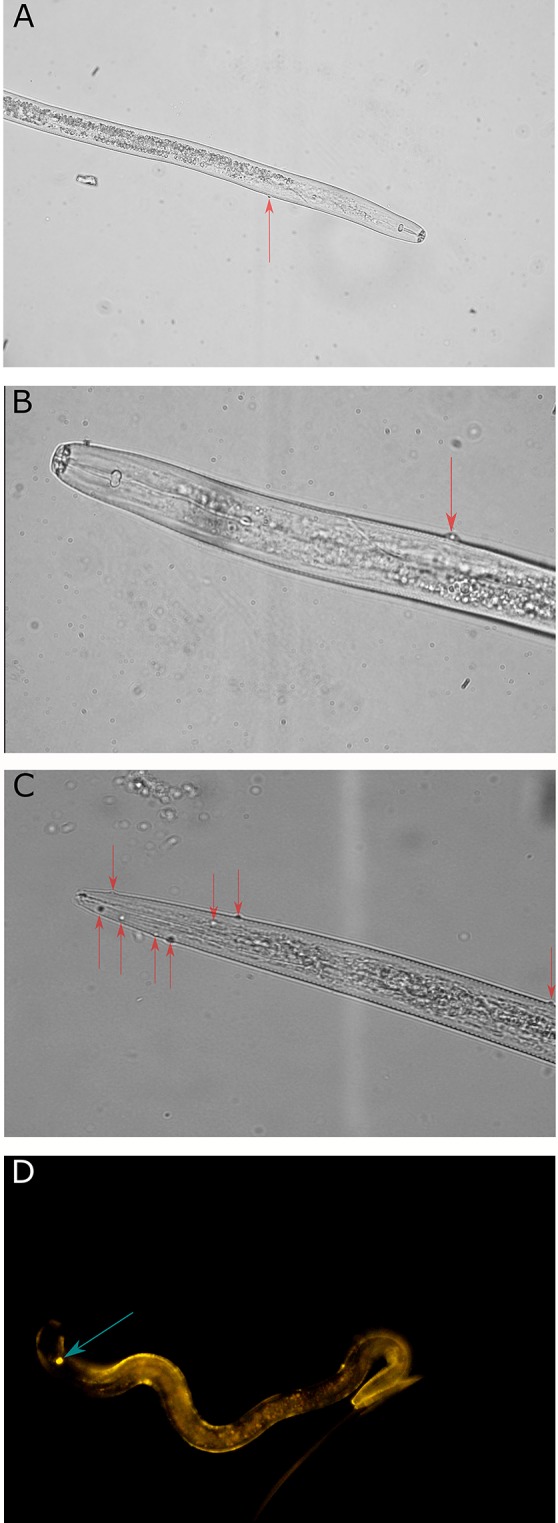
**(A)** 400x magnification *Pratylenchus* spp. recovered from ESFN soil with *Pasteuria* spp. endospore attached (position indicated by red arrow). **(B)** 1000x magnification of *Pratylenchus* spp. recovered from ESFN soil with *Pasteuria* spp. endospore attached (position indicated by red arrow). **(C)** 1000x magnification of free living non-parasitic nematode recovered from ESFN soil with several *Pasteuria* spp. endospores attached (positions indicated by red arrows). **(D)** 1000x magnification of fluorescence image of *Pratylenchus* spp. recovered from ESFN soil with *Pasteuria* spp. endospore attached (position indicated by blue arrow), showing anti-*Pasteuria penetrans* antibody recognition.

Sucrose floatation of nematodes from ESFN soils returned *Pasteuria* endospore encumbered *Pratylenchus* spp. and free-living nematodes (**Figure 6**). Insufficient material was recovered to amplify endospore 16S rRNA gene sequences, however polyclonal anti-*Pasteuria penetrans* antibodies successfully recognized *Pratylenchus* spp. attached endospores (**Figure 6**).

## Discussion

### Sensitivity

Sensitivity of our tests were high with detection limits of *Pasteuria* ranging from 10 to 1,000 copies of the target gene. Mauchline et al. (2011) hypothesized that the copy number of the 16S rRNA gene in *Pasteuria* spp. was likely to be low, one or two copies in contrast to those of related *Bacillus* spp., which typically have more than ten (Fogel et al., 1999). Genomic sequencing of *P. penetrans* RSE148 (Orr et al., manuscript in preparation) returned three SSU gene copies. Detection at this level would be theoretically sufficient to recover sequence from a single endospore filled juvenile nematode, which would typically contain ~500 mature endospores (Sturhan et al., 1994). These limits provide an indication of detection in an ideal sample where all bacteria are lysed, and which is free from inhibitors. However, spores of *Pasteuria* spp. are robust, being resistant or partially resistant to heat, desiccation, lysozyme, and SDS (Giannakou et al., 1997; Atibalentja et al., 2004; Mauchline et al., 2010). Environmental DNA samples are likely to contain inhibitors which would further increase the practical limits of detection relative to the inhibitor concentration within the starting material (Donn et al., 2008).

### Primer Specificity

UNOISE2 resulted in 148 16S rRNA gene ZOTUs across the entire dataset. Of these ZOTUs 75 (50.7%) were at least 90% similar to curated *Pasteuria* spp. reference sequences and only one sequence returned a best hit as low as 92% similarity. However, 100% of merged read pairs in the ESFN sample set and 93% of merged read pairs in the NSIS2 dataset were at least 90% identical to *Pasteuria* spp. reference sequences. The remaining 7% of NSIS2 assembled read pairs align predominantly with uncultured Acidobacteria. The lack of merged read pairs not matching to reference sequences suggests minimal off-target amplification from eDNA samples.

Nematode primers used in this study were not completely nematode specific, however ESFN DNA samples had been enriched for nematodes *via* Baermann funnel extraction. Nematode ZOTUs represented 581 (72%) of 803 18S rRNA gene ZOTUs and 85% of assembled read pairs illustrating that Baermann funnel extraction was an appropriate enrichment method. Sapkota and Nicolaisen (2015) reported a similar metabarcode study which enriched for nematode PCR products *via* initial semi-nested amplification using primers NemF and 18Sr2b from whole soil extracted DNA. These authors reported that 64.4% of OTUs recovered were taxonomically assigned to Nematoda (Sapkota and Nicolaisen, 2015). When contrasted with our results, PCR-based sample enrichment from whole soil extracted DNA appears to be less efficient (7.6% fewer nematode ZOTUs) than selection by Baermann funnel extraction. Similarly, *Pasteuria* spp. were amplified from a slightly higher proportion (45.9%) of ESFN samples compared to NSIS2 samples (35.9%), a difference of 7%. This slight increase in ESFN samples where *Pasteuria* spp. were detectable may be attributed to the greater volume of soil (200 g vs 0.25 g) which served as starting material for ESFN extractions. *Pasteuria* spp. may not be evenly distributed in soils and indeed the formation of microsites in soil may be important to effective parasitism (Stirling, 2014). Nematode enrichment from a sufficient volume of soil may also serve to enrich for *Pasteuria* species. Further, nested PCR reactions significantly increase the potential for well to well contamination, particularly within large sample sets. However, the proportional increase in *Pasteuria* spp. and nematode recovery in nematode enriched ESFN DNA extractions is slight; evaluating any statistical significance is not in the scope of this study. Further, it has been shown that an initial nested PCR reaction followed by a short number of barcoded primer cycles can reduce barcode bias effects and improve reproducibility in metabarcode studies (Berry et al., 2011).

### Ordination and Soil Properties

The physical and chemical properties of soils had clear and statistically significant influence on *Pasteuria* spp. community structure, moreover, *Pasteuria* ZOTUs had separate and specific relationships with these properties. *Pasteuria hartismeri* appears to significantly associate with mineral A horizons in the NSIS2 soil DNA sample set. Soil organic carbon, pH, moisture, and bulk density are intrinsically linked (Vereecken et al., 1989; Kemmitt et al., 2006). Soil pH has previously been shown to correlate well with bacterial diversity and elevation (Shen et al., 2013). In Scotland, this relationship has a clear geographic implication in that altitude, rainfall, and pH vary significantly from North and West to South and East. No significant correlations were observed with either latitude or longitude. However, *Pasteuria* spp. communities in the ESFN appear broadly similar, being dominated by *P. hartismeri* and *Pasteuria* Luffness sequences. Soil pH has also been shown to directly affect attachment of *P. penetrans* to root knot nematodes (Afolabi et al., 1995). *Pasteuria* spp. found parasitizing *H. glycines* in China were found most commonly in high pH, low organic matter soils (Ma et al., 2005). In isolation the observed relationships between *Pasteuria* spp. and soil properties could be perceived as factors affecting the survival, attachment, and retention of endospores. However, it is likely that the single greatest influence on the abundance and diversity of *Pasteuria* spp. is the distribution of their hosts. Near surface mineral horizons are the location in the soil profile most likely to contain recorded root feeding nematode hosts for *P. hartismeri*, temperate *Meloidogyne* species (Bishop et al., 2007). Bulk density, pH, and organic matter content were also shown by Treonis et al. (2018) to have a statistically significant effect on nematode community structure in parallel metabarcode and morphological profiling within grain production systems in the mid-Atlantic USA. These researchers demonstrated that PNNs, likely hosts for *P. hartismeri*, were more abundant in conventional and zero tillage systems compared to organic farms (Treonis et al., 2018). Our findings in the ESFN nematode community structure dataset broadly support those of Treonis et al. (2018) except for pH which shows no statistically significant influence either before or after Benjamini-Hochberg error correction in the ESFN dataset. This is likely a reflection of the limited range of pH within this sample set (5.1–6.6).

### *Pasteuria*-Nematode Community Relationships

Observed nematode-*Pasteuria* ZOTU correlations were not strong. This may be explained in part by successful nematode suppression. Where *Pasteuria* spp. are effectively parasitizing a nematode host, they may be difficult to recover as the number of juveniles in the community will be greatly reduced. They would also be less mobile due to the attachment of endospores to the cuticle, decreasing their recovery *via* methods which rely on the mobility of viable nematodes (Vagelas et al., 2012) and they may possess a much higher specific gravity, reducing their recovery *via* methods which rely on floatation (Oostendorp et al., 1991). Thus to successfully detect *Pasteuria* spp. from ESFN DNA samples both the bacteria and nematode had to be present; the nematode had to comprise enough of the total extracted metazoan community to detect; and the *Pasteuria* spp. had to be abundant enough to detect but not so abundant that the nematode could not be recovered. Despite this, the data provides some indication of *Pasteuria* spp. suppressive activity.

In ZOTU fits to the ESFN NMDS plots a *P. hartismeri*-like sequence which was prevalent in both datasets appeared to correlate negatively with almost all significant nematode species, including *Globodera* and *Pratylenchoides*. Suppression of *H. avenae* in UK soils by *Pasteuria-*like species has previously been reported (Davies et al., 1990). The dominance of *P. hartismeri* in Scottish soils is surprising. However, pairwise Spearman's rank correlations suggest a potential host range which is broader than it's currently described temperate RKN hosts (Bishop et al., 2007). Further, ZOTU X5, which is assigned with 100% identity to *Meloidogyne* spp. with exact matches to both *M. chitwoodi* and *M. fallax* with 99.7% identity to *M. minor* has a read abundance µl^−1^ ≥ 1 in 9.2% of successfully amplified ESFN samples. Fleming et al. (2016) recently surveyed cereal and grassland soils in Northern Ireland, finding that *M. minor* was prevalent in 6% of soils tested. The abundance of this ZOTU is, however, typically low. This may reflect both primer bias and an insufficient depth of sequencing. Waeyenberge et al. (2019) recently demonstrated that *M. incognita* are up to 19.3 x underrepresented in metabarcoding data of defined communities depending on method of DNA extraction and primer selectionRecovery of endospore encumbered *Pratylenchus* spp. suggests that the *P*. *hartismeri*-like *Pasteuria* found in this study is capable of attachment to Pratylenchinae as well as temperate RKN species. *P. hartismeri* assigned ZOTU X4 has a significant negative Spearman's rank correlation (P = 0.007) with *Pratylenchus fallax* although this relationship is not significant post Benjamini-Hochberg correction. A number of other nematode ZOTUs also demonstrate a statistically significant negative correlation with this *P. hartismeri*-like sequence including ZOTUs assigned to *Heterodera* spp. and *Paratylenchus* species. *Pasteuria* spp. are broadly considered to be extremely fastidious in their attachment profile, however, cross-superfamily attachment of *P*. *penetrans* to *Meloidogyne* spp. and *Pratylenchus* spp. has been reported (Oostendorp et al., 1990; Sharma and Davies, 1996). De Gives et al. (1999) tested the attachment of five *Pasteuria* endospore isolates to a range of nematodes finding that, while the attachment profile of two isolates from *M. incognita* were fastidious within RKN, isolates from three *Heterodera* spp. were less stringent; attaching to populations of *Heterodera* spp., *Globodera* spp., *M. javanica*, *Pratylenchus* spp., *Aphelenchoides* spp.*, Radopholus similis*, and *Rotylenchus reniformis*. Supporting this observation, Wishart et al. (2004) reported attachment of *P*. *nishizawae* endospores isolated from *H. glycines* to both *M*. *hapla* and *M*. *incognita*. Chen and Dickson (1998) listed 196 nematode species including free-living, predacious, plant-parasitic, and entomopathogenic nematodes which have been described encumbered with *Pasteuria* spp. endospores. However, the genetic diversity of *Pasteuria* spp. sequenced to date is relatively low. Mixed populations of *Pasteuria* spp. are often recorded (Davies et al., 1990), however, it is not often established that the endospores attached to each nematode species are distinct. Metabarcoding revealed surprisingly little genetic diversity of *Pasteuria* spp. in Scottish soils with two ZOTU sequences dominating in both agricultural and non-agricultural soils. However, we have captured only a fragment of the target gene, leaving the possibility that additional variability within these dominant ZOTU sequences has been overlooked. The range of nematodes to which attachment of well characterized *Pasteuria* spp. endospores has been tested is limited, normally restricted to root knot and cyst nematodes due to the focus on recovery of novel strains with biocontrol potential. While our understanding of the molecular mechanics of *Pasteuria* spp. and nematode surface coat interactions are incomplete, it may be premature to overstate the specificity of endospore attachment. Endospore attachment is one of the two mechanisms by which *Pasteuria* spp. may suppress a nematode population (Davies et al., 1991; Vagelas et al., 2012). Yet, in some cases, endospores attach and are then unable to germinate such as Noel et al. (2005) reported with *P. nishizawae* and *G. pallida*. This may account for indications of broad suppressive activity of our *P. hartismeri*-like sequence.

The near exact opposition of this *P. hartismeri*-like sequence and both *Catenaria*-like and *Pythium*-like sequences in NMDS ordination could indicate a competitive interaction for the same niche. *Catenaria* spp. are facultative parasites of a number of free living and PNNs (Stirling, 2014). *Pythium myophilum* (previously *Lagenidium myophilum*), the best taxonomic hit for ZOTU X88 is a parasite of shrimp (Nakamura et al., 1994). However, *Pythium* spp. have been recovered infecting of *Daphnia longispina* in several German lakes whose 18S rRNA gene sequence clustered with *P. myophilum* (Wolinska et al., 2009). *Daphnia* spp. are the only other recorded hosts outside of Nematoda for *Pasteuria* species (Duneau et al., 2011). Further, *Pythium monospermum*, a member of the same clade (Uzuhashi et al., 2010) is capable of parasitizing nematode eggs but not juveniles (Tzean and Estey, 1981; Wolinska et al., 2009; Duneau et al., 2011).

### Limitations and Opportunities for Future Development

Although correlated, direct PCN J2 counts were not accurately reflected in the abundance of merged read pairs assigned to *Globodera* species. Over and under estimation of nematode abundance has been widely reported in nematode metabarcoding studies (Porazinska et al., 2009; Treonis et al., 2018; Waeyenberge et al., 2019). In the case of Heteroderinae, this may have been exacerbated by the presence of a single nucleotide mismatch in the NF1 primer sequence to the target DNA. Sample collection, enrichment, DNA extraction, storage, barcoding, amplification, library preparation, and sequencing each introduce unique biases which can be minimized but are difficult to eliminate (Waeyenberge et al., 2019). Pending further practical and/or computational developments, it is important to recognize that sequence variant analysis of nematode communities remains largely indicative; not conclusive. Yet, several of the indicative relationships within the ESFN dataset corroborate previous literature (Treonis et al., 2018), or are corroborated in further investigation (**Figure 6**). This shows that, despite its limitations, metabarcoding can be a useful tool in uncovering biologically relevant trends in nematode communities.

The PCR barcoding strategy could be improved by double indexing of samples. This would account for possible primer dropout and allow for duplication of the assay in all samples. Alternatively, adapter indexes could be used (Glenn et al., 2016) which would preserve sequencing read length for PCR products. Waeyenberge et al. (2019) demonstrated that an adapted Holterman method (Holterman et al., 2006) offered improvements to complete lysis of nematode communities during DNA extraction. The use of multiple marker genes could provide a more robust method which is better able to discriminate taxa. Porazinska et al. (2009) found that sequencing of both 28S and 18S rDNA markers increased nematode taxon discrimination from 90% to 97% in complex artificial communities. However, an additional marker may reduce coverage or push up sequencing costs. Design of additional *Pasteuria* spp. markers is inhibited by a lack of robust sequence data from well characterized strains. Mauchline et al. (2010) identified several candidate markers such as *gyrB* and s*po0A* genes. A concerted effort to generate *Pasteuria* spp. reference sequences from diverse hosts and environments would be beneficial to understanding the true diversity of the genus and its evolutionary history. Further, conducting this assay in two stages, first assaying *Pasteuria* spp. populations from direct soil extracted DNA and then conducting deep sequencing of nematode communities, extracted using high density sucrose flotation, in a restricted number of interesting samples, may overcome some of the complications introduced by sampling strategy discussed above.

The influence of pH, bulk density, moisture, cation valence, and organic matter content on *P. penetrans* retention in the soil is well documented (Dabiré et al., 2001; Dabiré and Mateille, 2004; Dabiré et al., 2007; Mateille et al., 2009; Mateille et al., 2010). In addition, we have been able to show that these relationships may be species, or strain, specific and are likely driven by their effect on the host nematode. This method could also be used to conduct large scale assays of the effects of soil management practices in greater detail, such as investigation of tillage and organic amendment, on the cultivation of *Pasteuria* spp. specific suppression. Such an investigation may reveal a pathway to a more holistic integrated pest management strategy of which *Pasteuria* spp. are a part.

## Data Availability Statement

All code used to parse, analyze, and plot data presented in this article is available at: https://github.com/BioJNO/Pasteuria_Nematode_metabarcoding.

Paired end Illumina MiSeq data uploaded to the European Nucleotide Archive. Study accession: PRJEB34559.

Supporting data tables available on figshare: https://doi.org/10.6084/m9.figshare.9897374.

Figures available on figshare: https://doi.org/10.6084/m9.figshare.9849863.

## Author Contributions

JO, VB, PC, KD, RN, and TF contributed to the conception and design of the study. DR and TF were responsible for ESFN and NSIS2 DNA extractions respectively. JO carried out all other laboratory work and wrote the manuscript. JO and PC conducted all bioinformatic and statistical analyses. All authors contributed to manuscript revision, read, and approved the submission.

## Funding

This work is part of a Biotechnology and Biological Sciences Research Council Collaborative Award in Science and Engineering (BBSRC CASE) studentship with the James Hutton Institute, the University of Hertfordshire, and Syngenta (BB/M503101). The James Hutton Institute receives funding from the Scottish Government.

## Conflict of Interest

The authors declare that the research was conducted in the absence of any commercial or financial relationships that could be construed as a potential conflict of interest.
